# *Streptococcus pneumoniae* Impairs Maturation of Human Dendritic Cells and Consequent Activation of CD4^+^ T Cells via Pneumolysin

**DOI:** 10.1159/000522339

**Published:** 2022-03-04

**Authors:** Antje D. Paulikat, Lea A. Tölken, Lana H. Jachmann, Gerhard Burchhardt, Sven Hammerschmidt, Nikolai Siemens

**Affiliations:** Department of Molecular Genetics and Infection Biology, University of Greifswald, Greifswald, Germany

**Keywords:** Dendritic cells, *Streptococcus pneumoniae*, *Staphylococcus aureus*, Influenza A virus, Pneumolysin

## Abstract

Influenza A Virus (IAV), *Staphylococcus aureus* (staphylococci), and *Streptococcus pneumoniae* (pneumococci) are leading viral and bacterial causes of pneumonia. Dendritic cells (DCs) are present in the lower respiratory tract. They are characterized by low expression of co-stimulatory molecules, including CD80 and CD86 and high capacity of antigen uptake. Subsequently, DCs upregulate co-stimulatory signals and cytokine secretion to effectively induce T-cell priming. Here, we investigated these processes in response to bacterial and viral single as well as coinfections using human monocyte-derived (mo)DCs. Irrespective of single or coinfections, moDCs matured in response to IAV and/or staphylococcal infections, secreted a wide range of cytokines, and activated CD4<sup>+</sup>, CD8<sup>+</sup> as well as double-negative T cells. In contrast, pneumococcal single and coinfections impaired moDC maturation, which was characterized by low expression of CD80 and CD86, downregulated expression of CD40, and a mild cytokine release resulting in abrogated CD4<sup>+</sup> T-cell activation. These actions were attributed to the cholesterol-dependent cytotoxin pneumolysin (Ply). Infections with a *ply*-deficient mutant resulted in restored moDC maturation and exclusive CD4<sup>+</sup> T-cell activation. These findings show that Ply has important immunomodulatory functions, supporting further investigations in specific modalities of Ply-DC interplay.

## Introduction

The human respiratory tract is constantly exposed to a variety of environmental stimuli. Most of these stimuli are harmless. However, in case of encountering bacterial or viral pathogens, an immediate immune reaction is crucial for clearing the infection. This task is fulfilled by dendritic cells (DCs) among other cell types of myeloid lineage, one of the most efficient antigen-presenting cells (APCs) [1, 2, 3]. DCs exist in two distinct functional stages; immature and mature DCs. Immature DCs act as sentinels specialized in antigen uptake, facilitated by receptor-mediated endocytosis [4, 5, 6] and/or micropinocytosis [7], and antigen processing. Subsequently, DCs migrate from peripheral tissues to secondary lymphoid organs and undergo a maturation process. During this process, their ability to ingest additional antigens decreases. However, mature DCs are characterized by an increased production of cytokines and expression of (co-)stimulatory surface molecules, including major histocompatibility complex (MHC) class I and II as well as the cluster of differentiation (CD)80, CD86, and CD40 [3, 8].

Both CD80 and CD86 are closely related B7 receptors, which interact with the co-stimulatory receptor CD28 and the inhibitory cytotoxic T-lymphocyte-associated protein 4 on T cells [9]. A full T-cell activation is achieved via three different signals. First, the T-cell receptor interacts with the peptide-loaded MHCI and MHCII molecules on DCs. In a second step, B7 receptors interact with co-receptors on T cells. Finally, mature DCs release cytokines that shape the T-cell differentiation into distinct T helper cell subsets [3, 8, 10, 11]. During activation, T cells rapidly upregulate the expression of CD69, a known leukocyte activation marker [12]. In addition to B7 receptors, DCs express CD40, which is a transmembrane glycoprotein surface receptor. CD40 binds to CD40 Ligand on T cells resulting in a more effective activation of DCs by maintaining the expression of MHCII and additional upregulation of CD80 and CD86 [13].

One of the major causes of human respiratory tract infections is influenza A virus (IAV) [14]. Furthermore, *Staphylococcus aureus* (staphylococci) and *Streptococcus pneumoniae* (pneumococci), which are frequent colonizers of the upper respiratory tract, can cause severe respiratory diseases such as pneumonia, including community-acquired pneumonia (CAP) [15, 16, 17, 18, 19]. Studies on influenza pandemics have shown that IAV has the potential to synergize with these two Gram-positive pathogens. The so-called coinfections are characterized by an increased disease severity and higher mortality rates [20]. DCs are widely distributed throughout the human body and are localized in lymphoid as well as nonlymphoid tissues including mucosal surfaces of the respiratory tract. Therefore, DCs are of great importance in initiating an effective immune response against these three pathogens.

Staphylococci as well as pneumococci express a wide range of virulence factors including cytolysins. They lyse a variety of host cells in order to evade human immune response. Major cytolysins produced by *S. aureus* are α-hemolysin and the leukocidins Panton-Valentin leucocidin, LukAB, LukED, HlgAB, and HlgCB. Particularly, LukAB was shown to target human DCs via CD11b resulting in decreased activation of naïve T cells [21]. The pneumococcal pore-forming toxin pneumolysin (Ply) is a cholesterol-dependent cytolysin that forms ring-like pores in human cells and induces apoptosis [22]. At sublytic concentrations, Ply modulates the immune response by tempering with phagocytosis [23] or directly damaging immune effector cells [24]. It was shown that Ply inhibits the expression of co-stimulatory molecules on human DCs [25]. Furthermore, the mannose receptor C type 1 (MRC-1, CD206) was identified as a receptor for Ply. Ply-binding to MRC-1 inhibits toll-like receptor (TLR) signaling and production of pro-inflammatory cytokines by upregulation of suppressor of cytokine signalling 1 (SOCS1) [26].

In this study, we investigated the impact of IAV, *S. aureus,* and *S. pneumoniae* single as well as coinfections on monocytes-derived (mo)DC maturation and subsequent T-cell response. We show that irrespective of infection type, *S. aureus* and IAV activate moDCs. In contrast, pneumococcal infections result in impaired moDC maturation and consequently in an abrogated response by CD4^+^ T cells. This suppressive effect is mediated by Ply.

## Materials and Methods

### Bacterial and Viral Strains

*S. pneumoniae* TIGR4Δ*cps* and TIGR4Δ*cps*Δ*ply* mutant strains were generated as described previously [27]. Pneumococci were grown on blood agar plates (Oxoid) overnight and cultivated to mid-log phase (optical density 600 nm, 0.35–0.40) in Todd-Hewitt broth (Carl Roth) supplemented with 0.5% (w/v) yeast extract (Carl Roth) at 37°C and 5% CO_2_. *S. aureus* strain LUG2012 was grown overnight at 37°C in casein hydrolysate and yeast extract medium with agitation [28]. Influenza virus A/Bavaria/74/2009 (H1N1) was propagated as described previously [29].

### Isolation of Human Monocytes and PBMCs and moDC Generation

Human monocytes were isolated from buffy coats using CD14 S-pluriBead antihuman beads (PluriSelect) according to manufacturer's instructions. The moDCs were generated by culturing monocytes for 5 days in RPMI1640 (Cytivia) medium supplemented with 10% (v/v) heat inactivated fetal calf serum (FCS; Sigma-Aldrich, St. Louis, MO, USA), 89 ng × mL^−1^ GM-CSF and 22 ng × mL^−1^ interleukin (IL)-4 (both Immunotools). Medium was exchanged on day 3.

PBMCs were isolated from buffy coats via Lymphoprep density gradient centrifugation (Stemcell Technologies, Vancouver, BC, Canada). PBMCs were stored in FCS containing 10% (v/v) DMSO at −170°C until further analyses.

### DC Infections and CoCulture with PBMCs

All infections were performed in RPMI1640 complete media. 1 × 10^5^ monocyte-derived DCs (moDCs) were infected with H1N1 at a multiplicity of infection 0.1 for 24 h [30]. To warrant comparable initial bacterial infection rates, moDCs were infected at multiplicity of infection 10 for 3 h with pneumococci and 1 h with staphylococci. Next, the media was removed, and extracellular bacteria were killed by addition of RPMI1640 containing antibiotics (*S. aureus*: 400 μg × mL^−1^ gentamicin/2 μg × mL^−1^ lysostaphin (both Sigma-Aldrich); *S. pneumoniae*: 100 μg × mL^−1^ gentamicin/100 μg × mL^−1^ streptomycin/100 IU × mL^−1^ penicillin G (Hyclone). Coinfections combined viral and subsequent bacterial infection as described above. After infection, the moDCs were either directly prepared for flow cytometry or 1 × 10^6^ PBMCs (ratio of 10:1 based on uninfected control) were added for 3 day of coculture with subsequent flow cytometry analysis. For assessing intracellular bacterial survival kinetics, 2 × 10^5^ moDCs were infected as described above. After addition of antibiotics, the cells were washed, lysed, and intracellular bacteria plated on blood agar plates (Oxoid, Basingstoke, UK).

### Flow Cytometry

Dead cells were labeled using the Zombie Aqua Fixable Viability Kit (BioLegend, San Diego, CA, USA). Unspecific binding of immunoglobulins was blocked by using Human TruStain FcX (BioLegend) according to the manufacturer's instructions. Incubations of cells with titrated amounts of monoclonal antibodies were carried out for 30 min at 4°C in the dark. Cells were washed between each staining step and fixed using the Cyto-Fast Fix/Perm Buffer Set (BioLegend). Antibodies and clones directed against the following markers were used (target, clone, fluorochrome, all BioLegend): CD209 (9E9A8, APC), CD40 (5C3, Alexa Fluor700), CD80 (2D10, BV711), CD86 (BU63, PE/Cyanine7), MHCI (W6/32, Pacific Blue), MHCII (L243, FITC), CD3 (HIT3a, PerCP/Cyanine5.5), CD4 (SK3, KIRAVIA Blue 520), CD8 (QA18A37, PE), and CD69 (FN50, APC/Cyanine7). The respective gating strategies are shown in online supplementary Figure 1 (for all online suppl. material, see www.karger.com/doi/10.1159/000522339). Data were acquired using a FACSAriaIII flow cytometer and FACS DIVA 8.0 Software (both BD Biosciences, San Jose, CA, USA) and analyzed using FCS Express 7 Software (De Novo Software).

### Cytokine Measurements

Cytokine concentrations of infection supernatants were measured via LEGENDPlex human inflammation panel (13-plex) kit (BioLegend) according to the manufacturer's instructions. Data were acquired with a FACSAria III flow cytometer using FACS DIVA Software (both BD Bioscience) and analyzed using LEGENDPlex software (BioLegend).

### Viral Quantification

To quantify viral load in DCs, total RNA was isolated using the RiboPure RNA purification kit (Ambion, Austin, TX, USA) according to manufacturer's instructions. cDNA synthesis was performed using superscript first-strand synthesis system for RT-PCR (Invitrogen, Waltham, MA, USA). Quantitative RT-PCR amplification was performed with the SYBR GreenER Kit (BioRad, Hercules, MA, USA). The levels of β-Actin transcription were used for normalization. The following primers were used: nucleoprotein (NP) *NP*-for, 5′-TTCCACAAGAGGGGT −3′; *NP*-rev, 5′-TCCGTCCTTCACTGTTCC-3′; h-betaAct-for: 5′-CTCTTCCAGC­CTTCCTTCCT-3′; h-betaAct-rev, 5′-AGCACTGTGTTGGCGT­ACAG-3′.

### Statistics

Statistical significance of differences was determined using the Kruskal-Wallis test with Dunn's multiple comparison posttest. Statistics were performed using GraphPad Prism version 8. A *p* value less than 0.05 was considered significant.

## Results

### Impaired moDC Maturation in Response to Pneumococcal Infections

**Fig. 1:**
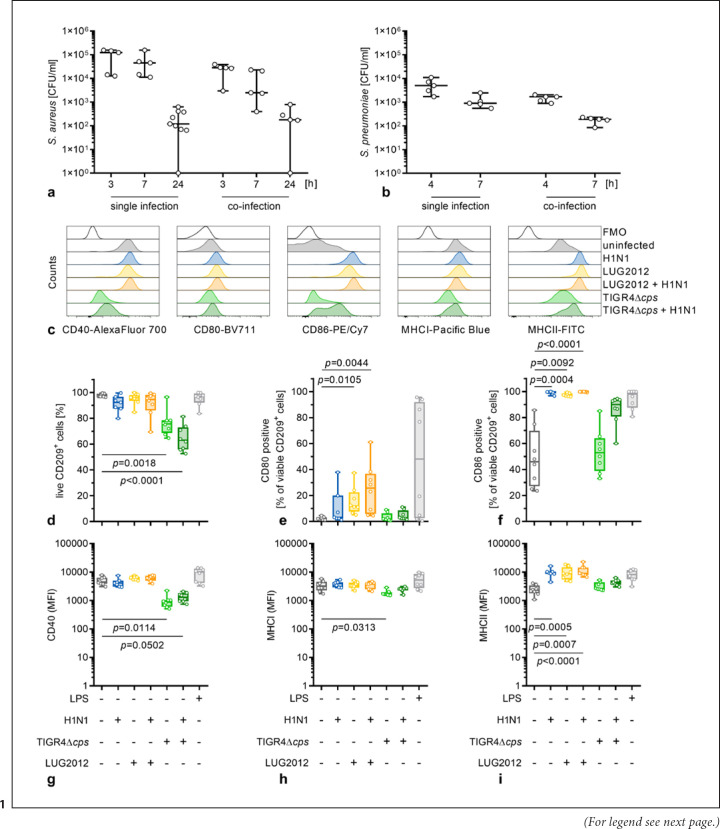
DC maturation is impaired in pneumococcal infections. Single and coinfections of moDCs with IAV (MOI 0.1), *S. aureus* LUG2012 (MOI 10), and *S. pneumoniae* TIGR4Δ*cps* (MOI 10) were performed. Extracellular staphylococci and pneumococci were killed by substituting the media with antibiotics. Viable intracellular staphylococci (**a**) and pneumococci (**b**) were determined at indicated time points (*n* ≥ 5). MoDC viability (**d**) and phenotype (**c, e–i**) were evaluated via flow cytometry (*n* ≥ 7). Representative histograms for each marker are shown in (**c**). The maturation process was evaluated assessing the frequencies of CD80^+^ (**e**) and CD86^+^ (**f**) cells as well as expression of CD40 (**g**), MHCI (**h**) and MHCII (**i**). Horizontal lines in (**a, b**) denote the median value with range. The data in (**d–i**) are displayed as box plots. Each dot represents one independent experiment with cells from one donor. The level of significance was determined using the Kruskal-Wallis test with Dunn's posttest. FMO, fluorescence minus one; MFI, mean fluorescence intensity; MOI, multiplicity of infection.

To investigate moDC maturation status in response to different infections, single infections of moDCs with H1N1, *S. pneumoniae* TIGR4Δ*cps,* and *S. aureus* LUG2012 or viral-bacterial coinfections were performed. First, viral replication in moDC was quantified. Irrespective of the infection type, approximately equal levels of *NP* RNA were detected in moDCs (online suppl. Fig. 2a). Next, the numbers of intracellular bacteria were determined over a period of up to 24 h. In contrast to pneumococci, staphylococci can persist in phagocytic cells. Our analyses confirmed this general observation. Although reduced numbers of both, LUG2012 and TIGR4Δ*cps* were recovered from moDCs over time (Fig. 1a, b), viable staphylococci were also found within the cells at t_24 h_. No difference in bacterial CFU was seen between single and coinfections (Fig. 1a, b).

Next, DC maturation was assessed via flow cytometry. Frequencies of CD40, CD80, CD86, MHCI, and MHCII positive cells as well as the expression level of these molecules were analyzed 24 h post infections (Fig. 1c-i and online suppl. Fig. 2). In general, mainly IAV and staphylococci-driven signatures were noted. H1N1 and LUG2012, alone or in combination, did not harm moDCs (Fig. 1d). Furthermore, H1N1 and LUG2012 single as well as coinfections resulted in increased frequencies of CD80^+^ and CD86^+^ moDCs (Fig. 1e, f). In addition, elevated expression levels of CD86 and MHCII were noted in response to these infections (online suppl. Fig. 2b and Fig. 1i). However, CD40, CD80, and MHCI expression remained unaffected (Fig. 1 and online suppl. Fig. 2). In contrast, moDC maturation was impaired in response to TIGR4Δ*cps* infections. Irrespective of the infection type, up to 40% of moDCs were killed by pneumococci (Fig. 1d). The expression levels of the majority of analyzed molecules as well as frequencies of cells expressing these molecules remained at the level of the uninfected control (Fig. 1 and online suppl. Fig 2). In contrast, reduced CD40 expression by moDCs was observed in all pneumococcal infections (Fig. 1g).

**Fig. 2:**
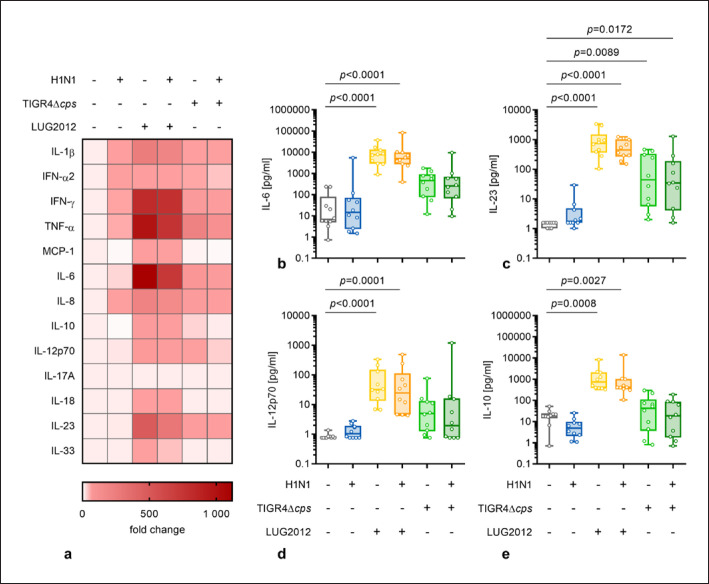
Cytokine secretion by moDC in response to infections. Cytokine secretion of infected moDCs was measured via a multiplex assay (*n* = 10). The heat map represents the fold change of cytokine concentration in relation to the uninfected control (**a**). Original data are displayed in online supplementary Figure 3 and (**b–e**). The concentrations of IL-6 (**b**), IL-23 (**c**), IL-12p70 (**d**) and IL-10 (**e**) was measured in supernatants of (un)infected moDCs. The data in (**b–e**) are displayed as box plots. Each dot represents one independent experiment with cells from one donor (*n* = 10). The level of significance was determined using the Kruskal-Wallis test with Dunn's posttest.

Mature moDCs produce cytokines to influence the subsequent adaptive immune response [11]. Therefore, multiple inflammatory cytokines were measured in supernatants of infected moDCs. Overall, elevated levels of cytokine secretion, except for interleukin (IL)-17A, were noted in response to the majority of infections. However, significantly higher concentrations of cytokines, especially interferon (IFN)-γ, tumor necrosis factor (TNF)-α, IL-6, and IL-23, were measured mainly in infections involving staphylococci (Fig 2, online suppl. Fig. 3). Since the used H1N1 strain is of mild infectivity [31], no major cytokine secretion by moDCs was observed. Only IL-1β and IFN-α2 were detected in higher levels in response to the single viral infection as compared to uninfected controls (online suppl. Fig. 3e). Notably, minor alterations in cytokine secretion were detected in infections involving pneumococci. TNF-α, IFNs, and IL-23 were elevated in these infections, while the others remained mostly at the level of the uninfected control (Fig. 2 and online suppl. Fig. 3).

### Pneumococci-Infected moDCs Activate CD8^+^ and Double-Negative T Cells but Fail to Activate CD4^+^ T Cells

**Fig. 3:**
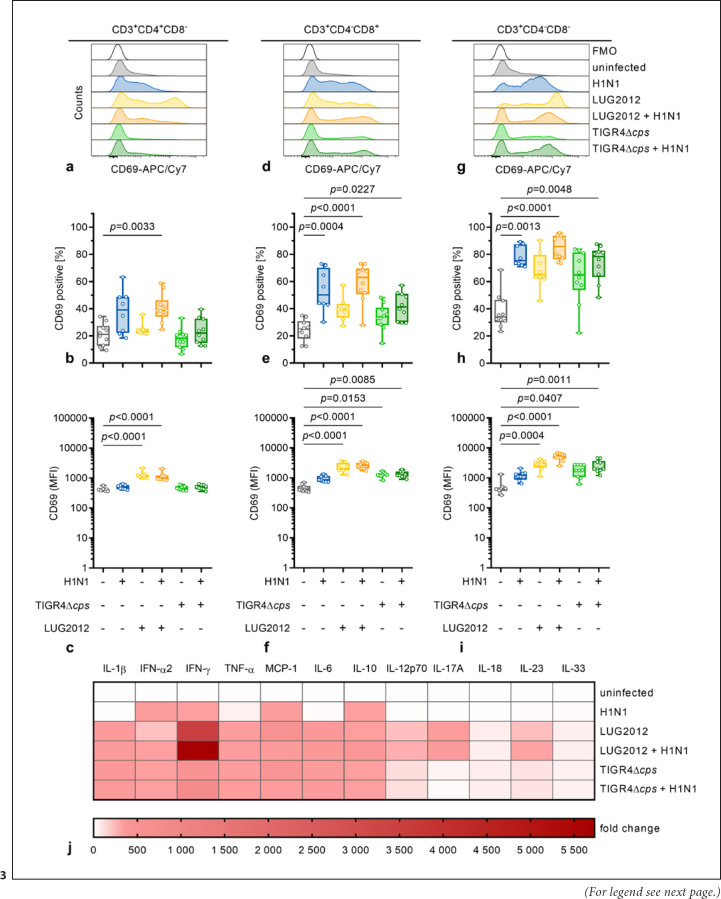
T-cell activation after cocultures with infected moDCs. Infected moDCs were cocultured with autologous PBMCs for 3 days and T cells were analyzed via flow cytometry (*n* ≥ 8). The activation of CD4^+^ (**a–c**), CD8^+^ (**d–f**), and DN (**g–i**) T cells was evaluated based on the expansion (**b, e, h**) and/or expression of CD69 (**c, f, i**). Representative histograms are shown in (**a, d, g**). **j**Cytokine secretion of PBMCs cocultured with infected moDCs. The heat map represents the fold change of cytokine concentration in relation to the uninfected control (*n* ≥ 8). Original data are displayed in online supplementary Figure 4. The data in (**b, c, e, f, h, i**) are displayed as box plots. Each dot represents one independent experiment with cells from one donor. The level of significance was determined using the Kruskal-Wallis test with Dunn's posttest. FMO, Fluorescence minus one; MFI, mean fluorescence intensity.

Surface molecule expression and cytokine secretion analyses indicated that moDCs mature in response to staphylococcal infections, while this process is partially inhibited in pneumococcal infections. Since DCs are crucial for orchestrating a specific T-cell response, we examined the capacity of infected moDCs to activate human T cells. Infected human moDCs were cocultured with autologous PBMCs for 3 d and T-cell activation as well as cytokine secretion were measured. Activation of T cells was defined by the expansion of CD69^+^ cell populations and/or increased expression of this molecule. These analyses revealed that H1N1 infected moDC (single and coinfected) activated CD8^+^ and double-negative (DN) T cells (Fig. 3). Staphylococci-infected moDCs activated all three T-cell subsets (CD4^+^, CD8^+^, DN; Fig. 3). In contrast, coculture of single pneumococci-infected moDCs with PBMCs did not result in expansion of CD69^+^ expressing T cells (Fig. 3). However, higher expression of CD69 in CD8^+^ and DN T cells was noted (Fig. 3). Furthermore, cytokines were measured in supernatants of the cocultures. Overall, coincubations of infected moDCs with PBMCs resulted in increased secretion of all measured cytokines as compared to the moDCs alone (Fig. 3j). Specifically, IFN-α2 and IL-10 release were high in single viral infections (online suppl. Fig. 4b, g). Staphylococci-infected moDCs caused significant secretion of all analyzed cytokines (Fig. 3 and online suppl. Fig. 4). This was also true for all pneumococcal infections, except for secretion of IL-17A (Fig. 3 and online suppl. Fig. 4).

### Ply Is Responsible for Diminished moDC Maturation and Consequently Impaired Activation of CD4^+^ T Cells

**Fig. 4:**
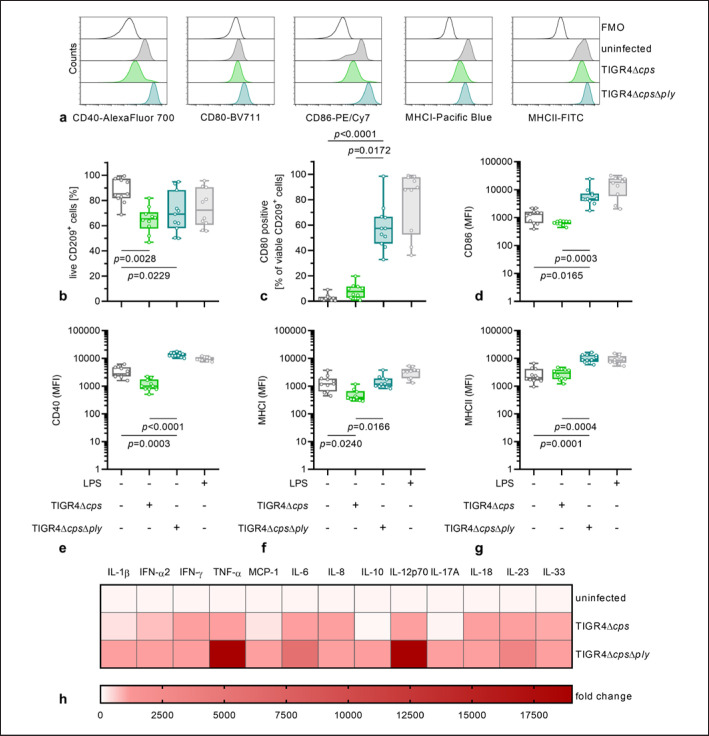
Ply impairs moDC maturation and cytokine secretion. MoDCs were infected with TIGR4Δ*cps* or the corresponding ply-deficient mutant (TIGR4Δ*cps*Δ*ply*) at MOI 10. Extracellular pneumococci were killed by substituting the media with antibiotics. MoDC viability (**b**) and phenotype (**c–g**) were evaluated via flow cytometry (*n* ≥ 10). Representative histograms for each marker are shown in (**a**). The maturation process was evaluated assessing the frequencies of CD80^+^ (**c**) cells as well as expression of CD86 (**d**), CD40 (**e**), MHCI (**f**), and MHCII (**g**). **h**Supernatants were collected and cytokine concentration was measured. The heat map represents the fold change of cytokine concentration in relation to the uninfected control. Original data are displayed in online supplementary Figure 6. The data in (**b–g**) are displayed as box plots. Each dot represents one independent experiment with cells from one donor (*n* ≥ 10). The level of significance was determined using Kruskal-Wallis test with Dunn's posttest. FMO, Fluorescence minus one; MFI, mean fluorescence intensity; MOI, multiplicity of infection.

Our analyses show that irrespective of single or coinfections, pneumococci inhibit moDC maturation and subsequent T-cell response. Ply was previously shown to bind to MRC-1, leading to suppressed pro-inflammatory cytokine secretion and TLR-signaling in human DCs [26]. To determine whether Ply inhibits maturation of moDCs, a series of proof-of-concept single pneumococcal infections was performed. Therefore, moDCs were infected with TIGR4Δ*cps* and the corresponding TIGR4Δ*cps*Δ*ply* strain. Both TIGR4Δ*cps*Δ*ply* and its parental TIGR4Δ*cps* strain showed comparable survival kinetics within moDCs (online suppl. Fig. 5a). Furthermore, no differences in moDC viability were noted. Both infections resulted in approximately 40% cell death (Fig. 4b). However, infection of moDCs with the *ply*-deficient strain resulted in expansion of CD80^+^ and CD86^+^ cells as well as in increased expression of CD86, CD40, and MHCII as compared to the uninfected or TIGR4Δ*cps*-infected moDCs (Fig. 4 and online suppl. Fig. 5). TIGR4Δ*cps* infections confirmed the previously observed suppressive moDC phenotype (Fig. 4 and online suppl. Fig. 5). In addition, cytokine secretion in response to both infections was analyzed. Again, only a mild cytokine secretion was noted in response to TIGR4Δ*cps* infections (Fig. 4h and online suppl. Fig. 6). In contrast, TIGR4Δ*cps*Δ*ply* infections of moDCs resulted in increased release of all analyzed cytokines (Fig. 4h and online suppl. Fig. 6). Particularly, IL-12p70, which directs Th1 differentiation, was exclusively released in response to TIGR4Δ*cps*Δ*ply* infections (Fig. 4h and online suppl. Fig. 6i).

**Fig. 5:**
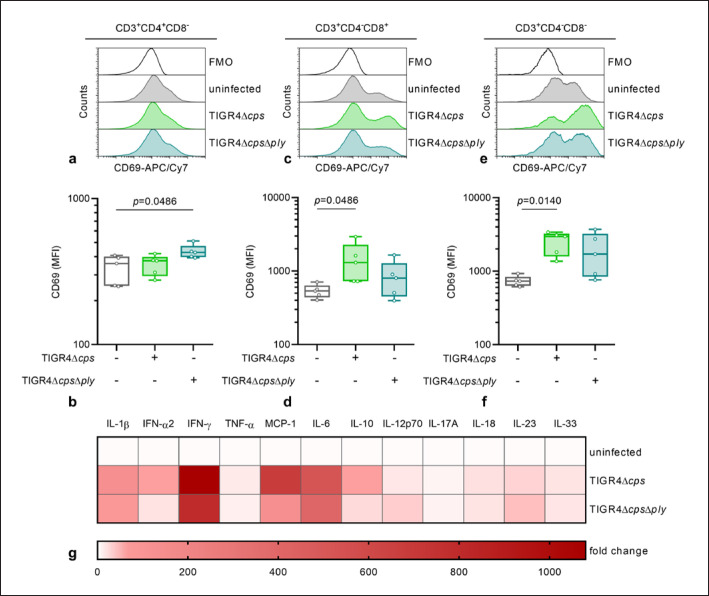
T-cell activation in response to cocultivation with moDCs infected with *ply*-deficient mutant strain. Infected moDCs were cocultured with PBMCs for 3 days and PBMCs were analysed via flow cytometry (*n* = 5). The activation of CD4^+^ (**a, b**), CD8^+^ (**c, d**), and DN (**e, f**) T cells was evaluated based on the expression of CD69 (**b, d, f**). Representative histograms are shown in (**a, c, e**). **g**Cytokine secretion of PBMCs cocultured with infected DCs was measured via a multiplex assay. The heat map represents the fold change of cytokine concentration in relation to the uninfected control. Original data are displayed in online supplementary Figure 8. The data in (**b, d, f**) are displayed as box plots. Each dot represents one independent experiment with cells from one donor (*n* = 5). The level of significance was determined using Kruskal-Wallis test with Dunn's posttest. FMO, fluorescence minus one; MFI, mean fluorescence intensity.

Next, infected moDCs were cocultured with autologous PBMCs and T-cell activation as well as the cytokine release was determined. Again, TIGR4Δ*cps* infected moDCs activated CD8^+^ and DN T cells (Fig. 5a-f, online suppl. Fig. 7). In contrast, TIGR4Δ*cps*Δ*ply* infected moDCs activated CD4^+^ T cells as shown by increased expression of CD69 (Fig. 5a, b, online suppl. Fig. 7). Analysis of cytokine release in cocultures showed no TIGR4Δ*cps*Δ*ply* specific signatures (Fig. 5 and online suppl. Fig. S8). However, particularly IFN-γ, monocyte chemoattractant protein-1, and IL-6 were detected in high amounts in both infectious conditions (Fig. 5 and online suppl. Fig. 8).

## Discussion/Conclusion

DCs are of great importance in initiating an immune response against respiratory pathogens, including *S. aureus, S. pneumoniae,* and IAV. They detect specific components of the pathogens, which leads to a maturation process. This process is characterized by an upregulation of (co-)stimulatory molecules in order to provide the important signals for activation of naïve T cells as well as secretion of the polarizing cytokines. Here, we show that *S. aureus* as well as IAV infections of moDCs induce a maturation process and subsequent specific T-cell activation. In contrast, pneumococcal infections result in an impaired moDC maturation and a moderate cytokine response. Subsequently, pneumococci-infected moDCs failed to activate CD4^+^ T cells. These immune cell responses were attributed to Ply. Infections of moDCs with Ply-deficient pneumococci restored the maturation process in moDCs resulting in activation of CD4^+^ T cells.

IAV is a major cause for respiratory tract infections and the development of CAP [14]. It was observed that certain IAV strains have the ability to impair DC maturation, especially the expression of co-stimulatory receptors CD80 and CD86 [32, 33]. Although an H1N1 strain from the 2009 pandemic was used, our results contradict previous observations. The influenza virus A/Bavaria/74/2009 induced moDC maturation resulting in activation of CD8^+^ and DN T cells. However, since this strain is of mild infectivity [31], the detected response was overall weak. *S. aureus* persistently colonizes the upper respiratory tract of approx. 30% of the population and in general, staphylococci are potent trigger of DC maturation and subsequent T-cell activation [34]. Staphylococcal virulence factors that interfere with DCs are LukAB and phenol soluble modulins, among others [21, 35, 36]. Berends et al. [21] have shown that LukAB potently kills human DCs resulting in reduced activation of CD4^+^ T cells. However, *S. aureus* used in this study remained silent within moDCs without major effects on the cell viability. Moreover, a clear maturation process and subsequent CD4^+^ T-cell activation was noted. In contrast, pneumococci, which also frequently colonize the upper respiratory tract and are the major cause of bacterial CAP [37, 38, 39], completely impaired moDC maturation and CD4^+^ T-cell activation in a Ply-dependent manner. Littmann et al. [25] have already demonstrated Ply-mediated suppression of CD80 and CD86 expression on human DCs by a yet unknown mechanism. Here, we show that CD40 expression on moDC is also affected by Ply.

Activated CD4+ T cells differentiate into different T helper subsets. This process is mainly shaped by cytokines secreted by DCs [11]. Th1 cells provide protection from intracellular pathogens, including viruses and invading bacteria. IL-12 and IL-27 induce Th1 polarization leading to an additional production of IL-12, TNFα, and IFNγ [40, 41, 42]. While staphylococcal as well as Ply-deficient pneumococcal infections induced moDC maturation and IL-12p70 release, single and coinfections with Ply-expressing TIGR4Δ*cps* pneumococci and IAV did not. These results are partially in line with previous studies. Spelmink et al. reported that IAV itself does not induces IL-12p70 but markedly upregulates TLR3 expression in moDCs. The subsequent phagocytosis and processing of pneumococci result in TLR3-dependent sensing of pneumococcal RNA, which is a key signal to drive IL-12p70 production and secretion [43, 44]. However, the mentioned study solely focused on RNA-mediated effects. Later, it was demonstrated that Ply binds to MRC-1 on DCs, which in turn results in upregulation of SOCS1 and ultimately leads to suppressed cytokine production and secretion [26]. Furthermore, IL-12p70 production is enhanced by CD40 (moDCs) and CD40 Ligand (T cells) interaction [45]. Our data show that pneumococcal infections result in downregulation of CD40 on moDCs potentially leading to reduced Th1 polarization of T cells. However, coculture of pneumococci-infected moDCs with PBMCs resulted in increased secretion of IL-12p70, TNFα, and IFNγ. Of note, (i) PBMCs were used in coculture experiments, which might mask true pneumococci-mediated effects through e.g., interaction of monocytes with T cells and (ii) a higher ratio of PBMCs:moDCs was present in pneumococcal infections due to the cytotoxic events toward moDCs, which might result in enhanced secretion of mentioned cytokines.

Th17 polarization of CD4^+^ T cells is another important defense mechanism against extracellular pathogens. The Th17 differentiation requires IL-6 and IL-23, while Th17 cells themselves produce IL-17 and IL-22 [41, 42]. It was shown that Th17 cells are of great importance for bacterial clearance at mucosal surfaces [40] and play an important role in protection against pneumococcal colonization in mice [46, 47]. While staphylococci-infected moDC released high amounts of the mentioned key cytokines and induced IL-17A release in coculture, this was not the case in pneumococcal infections. A recently published study showed that Th17 polarization positively correlates with high expression of CD40 on human DCs [48]. Although infections of moDCs with Ply-deficient pneumococci led to an upregulation of CD40 and elevated release of IL-6 and IL-23, IL-17A production was again abrogated in coculture experiments. Whether pneumococci possess the ability to suppress Th17 polarization warrants further studies.

Of note, bacteria-infected moDCs activated CD8^+^ and DN T cells. DCs are able to internalize, process, and present exogenous antigens via MHC class I, a process termed cross-presentation, which is particularly important for activation of CD8^+^ T cells [49]. A recent study demonstrated that MRC-1 is involved in enhanced cross-presentation activity of DCs through antigen localization in early endosomes [50]. Since it is known that Ply binds to MRC-1, we can only speculate that this interaction within moDCs might lead to enhanced cross-presentation and consequent activation of CD8^+^ T cells in pneumococcal infections. This fact is further supported by the observation that CD8^+^ T cells were activated by TIGR4Δcps-infected moDCs. This process was abrogated in infections with Ply-deficient mutant.

In conclusion, infections of moDC with IAV and/or *S. aureus* induced maturation and subsequent activation of all types of T cells. In contrast, pneumococcal infections blocked these processes through Ply-mediated suppression of co-stimulatory receptors CD40, DC80, and CD86. Particularly, CD4^+^ T-cell activation was completely abrogated. These effects were reversed by the use of Ply-deficient strain. Further studies to determine a more detailed role for pneumococci-DC interplay and their subsequent interaction with T cells are warranted.

## Statement of Ethics

Buffy coats obtained from healthy blood donors were anonymously provided by the blood bank at the University Medicine Greifswald. The use of PBMCs, monocytes for moDC generation, infections and coincubations with donor matched PBMCs was approved by the Ethical Research Committee at the University Medicine Greifswald (Ref. No. BB 014/14). All experiments were carried out in accordance with the approved guidelines.

## Conflict of Interest Statement

The authors have no conflicts of interest to declare.

## Funding Sources

This research was supported by the Federal Excellence Initiative of Mecklenburg Western Pomerania and European Social Fund Grant KoInfekt (ESF_14-BM-A55-0001_16 to S.H.) and the German Research Foundation (DFG; Grant No. 407176682 to N.S.).

## Author Contributions

A.D.P., L.A.T., S.H., and N.S. designed the study. A.D.P., L.A.T., L.H.J., and G.B. performed the experiments. A.D.P., L.A.T., L.H.J., and N.S. analyzed the data. A.D.P., L.A.T., and N.S. wrote the manuscript. All authors read, edited, and reviewed the manuscript.

## Data Availability Statement

All data generated during this study are included in this article and its online supplementary material files. Further inquiries can be directed to the corresponding author upon reasonable request.

## Supplementary Material

Supplementary data
