# Supplementary material for: Streptococcus pneumoniae Impairs Maturation of Human Dendritic Cells and Consequent Activation of CD4+ T Cells via Pneumolysin
Source: J Innate Immun. 2022 Mar 4;14(5):569–80. doi: 10.1159/000522339 (PMC9485967; doi:10.1159/000522339)
Supplement: Supplementary file 1 — Supplementary data [file jin-0014-0569-s01.pdf]

Supplementary Material

Streptococcus pneumoniae Impairs Maturation of Human Dendritic Cells and consequent activation of CD4$^+$ T cells via Pneumolysin

Antje D. Paulikat[a], Lea A. Tölken[a], Lana H. Jachmann[a], Gerhard Burchhardt[a], Sven Hammerschmidt[a], Nikolai Siemens[a]

[a] Department of Molecular Genetics and Infection Biology, University of Greifswald, Greifswald, Germany

Short Title: Pneumolysin impairs DC maturation

Corresponding Author:

Nikolai Siemens

University of Greifswald

Department of Molecular Genetics and Infection Biology

Felix-Hausdorff-Straße 8

D-17487 Greifswald, Germany

E-mail: nikolai.siemens@uni-greifswald.de

Phone: +49 (0) 3834 420 57 11

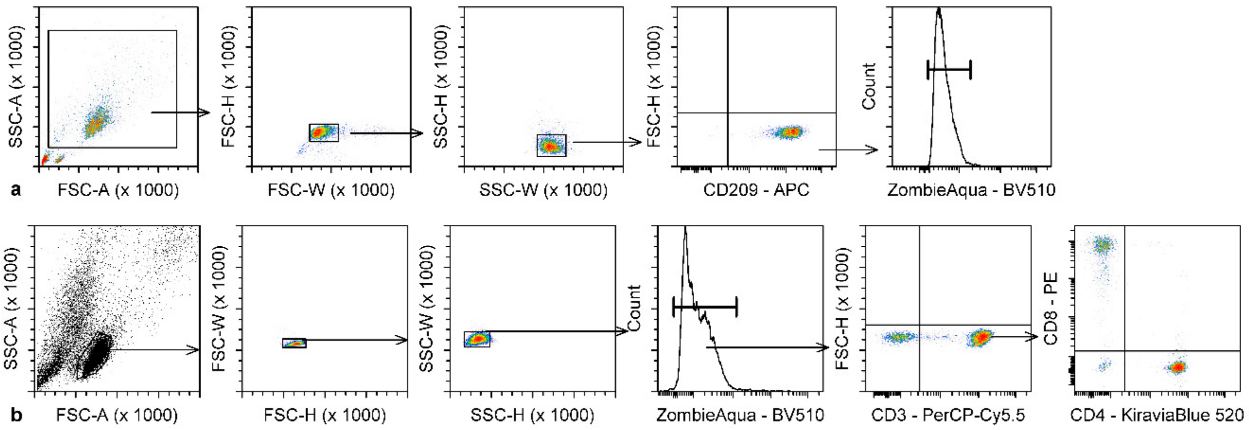

Figure S1 Gating strategy used to identify human moDCs (**a**) and T cells (**b**). Doublets were excluded by consecutive gating of FSC-H/FSC-W and SSC-H/SSC-W. MoDCs (**a**) were selected based on the expression of the specific DC marker DC-SIGN (CD209). Dead cells were excluded by using the Zombie Aqua™ Fixable Viability Kit. For the identification of T cells (**b**): first dead cells were excluded and T cells were selected based on the expression of CD3 and additionally identified as $CD8^+$ (upper left), $CD4^+$ (lower right) and double negative (lower left) T cells.

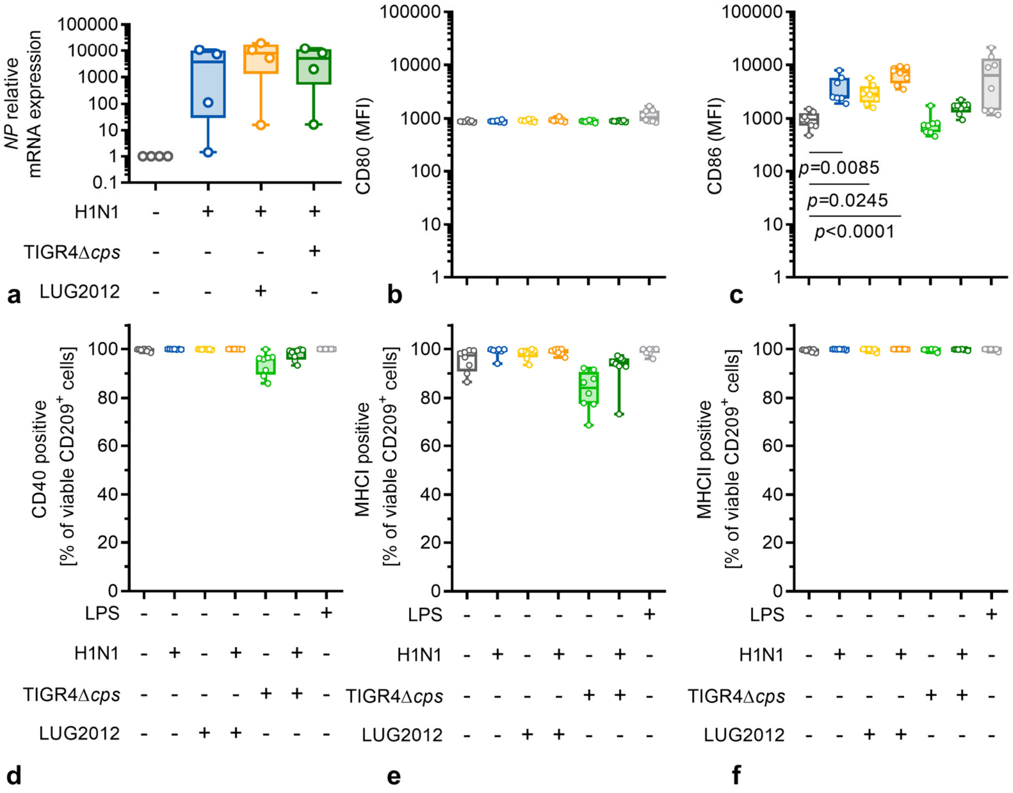

Figure S2 Verification of viral infection (**a**) and evaluation of DC maturation (**b-f**). Viral infection was validated by the relative *NP* mRNA expression (**a**) (n=4). Single and co-infections of moDCs with IAV (MOI 0.1), *S. aureus* LUG2012 (MOI 10) or *S. pneumoniae* TIGR4Δ*cps* (MOI 10) were performed (n≥7). Maturation was evaluated based on the expression of CD80 (**b**) and CD86 (**c**) as well as frequencies of CD40^{+} (**d**), MHCI^{+} (**e**) and MHCII^{+} (**f**) cells using flow cytometry. The data are displayed as box plots. The level of significance was determined using Kruskal-Wallis test with Dunn`s post-test. (MFI, mean fluorescence intensity).

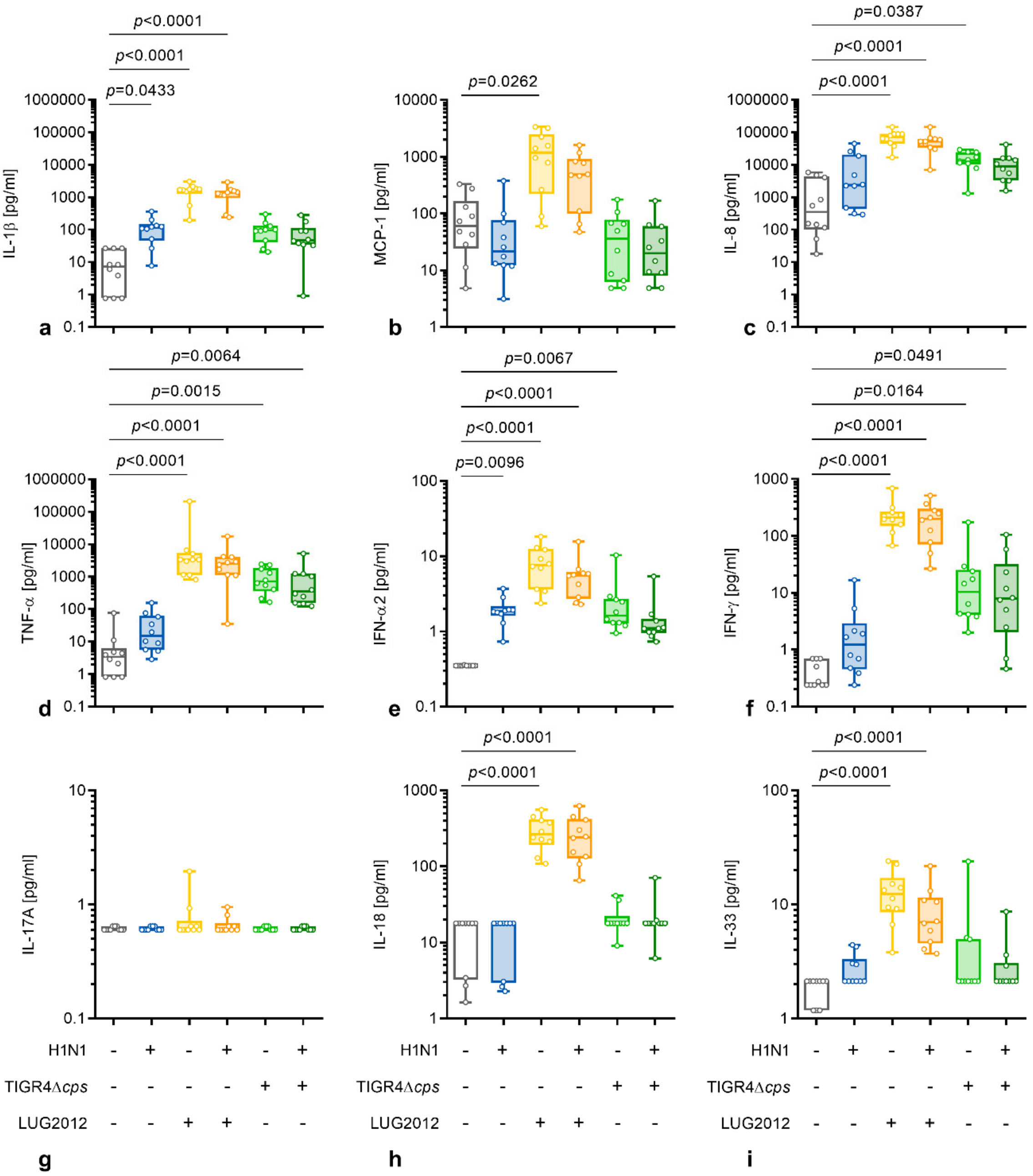

Figure S3 Cytokine secretion by moDCs in response to infections. Cytokine secretion of infected moDCs was measured via a multiplex assay (n=10). The concentration of IL-1β (**a**), MCP-1 (**b**), IL-8 (**c**), TNF-α (**d**), IFN-α2 (**e**), IFN-γ (**f**), IL-17A (**g**), IL-18 (**h**) and IL-33 (**i**) was measured in supernatants of (un)infected moDCs. The data are displayed as box plots. The level of significance was determined using Kruskal-Wallis test with Dunn`s post-test.

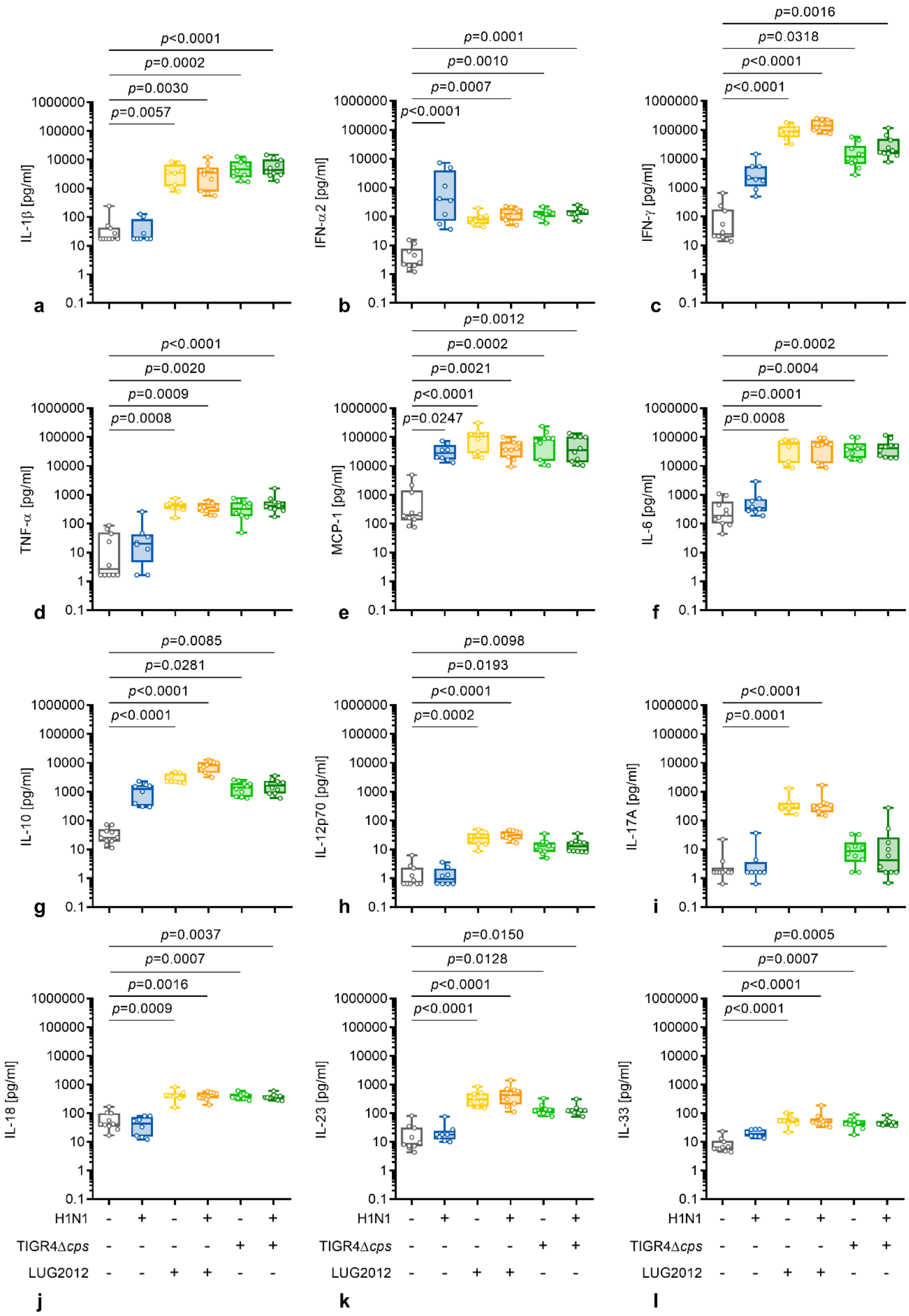

Figure S4 Cytokine secretion by moDC-PBMCs co-cultures. Cytokine secretion of PBMCs co-cultured with infected DCs was measured via a multiplex assay (n ≥ 8). The concentration of IL-1β (**a**), IFN-α2 (**b**), IFN-γ (**c**), TNF-α (**d**), MCP-1 (**e**), IL-6 (**f**), IL-10 (**g**), IL-12p70 (**h**), IL-17A (**i**), IL-18 (**j**), IL-23 (**k**) and IL-33 (**l**) was determined using flow cytometry. The data are displayed as box plots. The level of significance was determined using Kruskal-Wallis test with Dunn`s post-test.

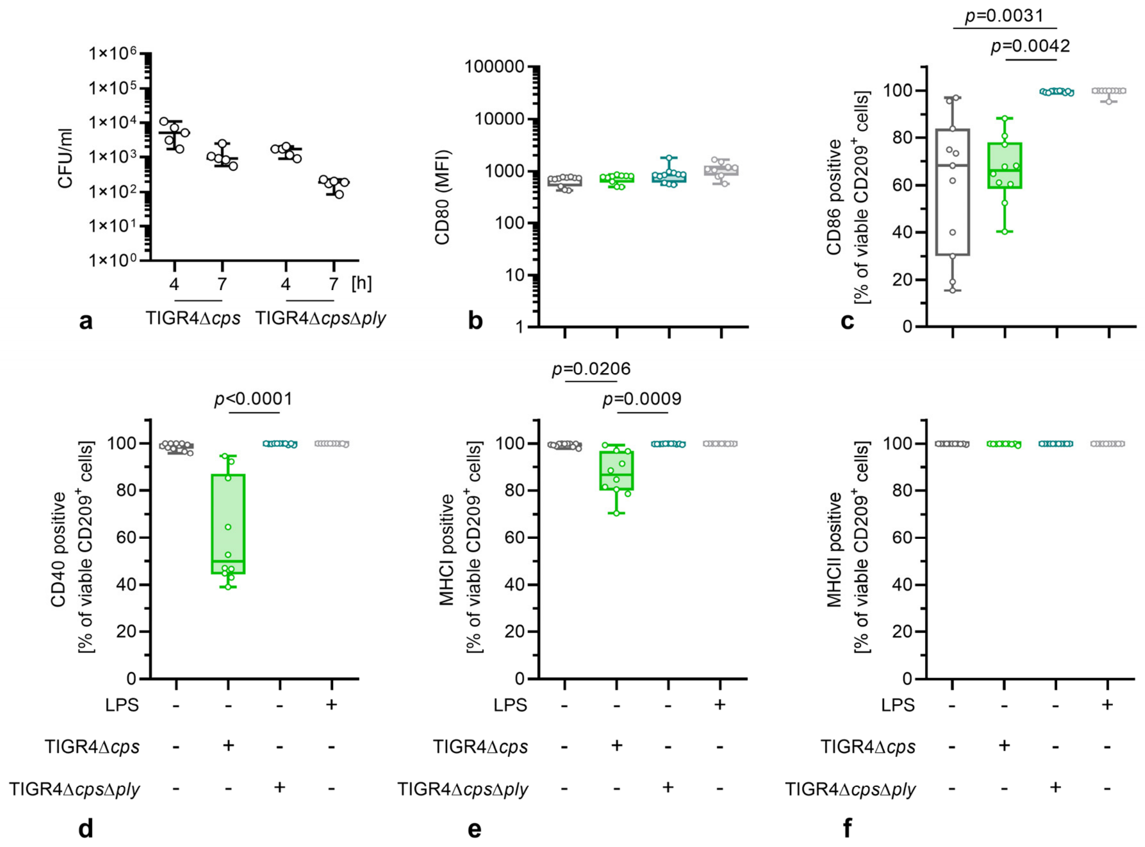

Figure S5 Impact of pneumolysin on dendritic cells. MoDCs were infected with TIGR4Δ*cps* or the corresponding Ply mutant (both MOI 10). Extracellular pneumococci were killed by substituting the media with antibiotics. Amounts of viable intracellular Bacteria were evaluated at indicated time points (**a**) (n ≥ 5). MoDC phenotype (**b-f**) was evaluated via flow cytometry (n≥10). The maturation process was evaluated assessing the expression of CD80 (**b**) as well as the frequencies of CD86^{+} (**e**), CD40^{+} (**d**), MHCI^{+} (**e**) and MHCII^{+} (**f**) cells. Horizontal lines (**a**) denote the median value with range. The data in (**b-f**) are displayed as box plots. The level of significance was determined using Kruskal-Wallis test with Dunn`s post-test. (MFI, mean fluorescence intensity).

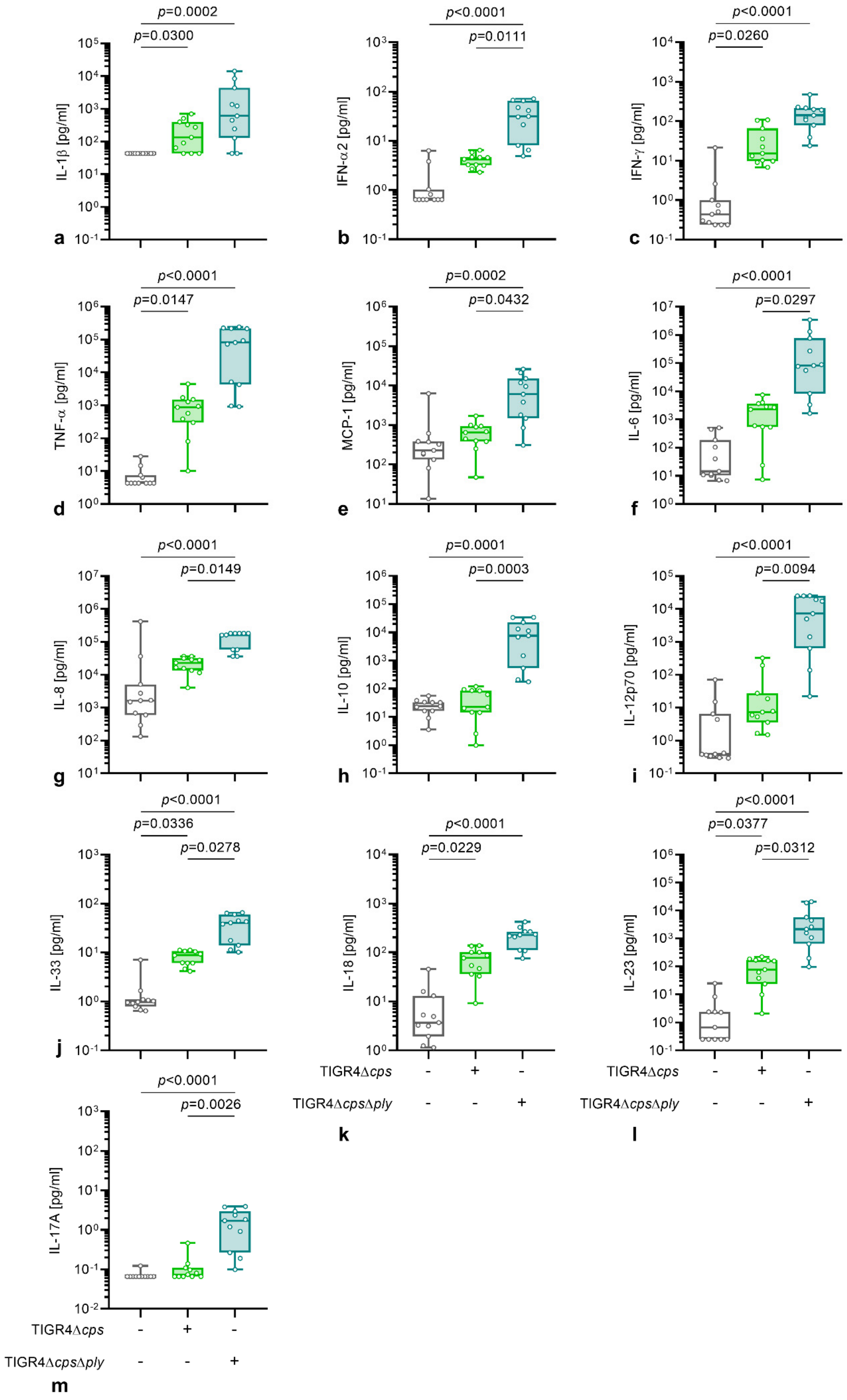
p=0.0002
p=0.0300
IL-1β [pg/ml]
a
p<0.0001
p=0.0111
IFN-α2 [pg/ml]
b
p<0.0001
p=0.0260
IFN-γ [pg/ml]
c
p<0.0001
p=0.0147
TNF-α [pg/ml]
d
p=0.0002
p=0.0432
MCP-1 [pg/ml]
e
p<0.0001
p=0.0297
IL-6 [pg/ml]
f
p<0.0001
p=0.0149
IL-8 [pg/ml]
g
p=0.0001
p=0.0003
IL-10 [pg/ml]
h
p<0.0001
p=0.0094
IL-12p70 [pg/ml]
i
p<0.0001
p=0.0336
p=0.0278
IL-33 [pg/ml]
j
p<0.0001
p=0.0229
IL-18 [pg/ml]
k
p<0.0001
p=0.0377
p=0.0312
IL-23 [pg/ml]
l
p<0.0001
p=0.0026
IL-17A [pg/ml]
m
TIGR4Δcps - + -
TIGR4ΔcpsΔply - - +

Figure S6 Cytokine secretion by moDCs in response to infections. Cytokine secretion of infected moDCs was measured via a multiplex assay (n=11). The concentration of IL-1β (**a**), IFN-α2 (**b**), IFN-γ (**c**), TNF-α (**d**), MCP-1 (**e**), IL-6 (**f**), IL-8 (**g**), IL-10 (**h**), IL-12p70 (**i**), IL-33 (**j**), IL-18 (**k**), IL-23 (**l**) and IL-17A (**m**) was measured in supernatants of (un)infected moDCs. The data are displayed as box plots. The level of significance was determined using Kruskal-Wallis test with Dunn`s post-test.

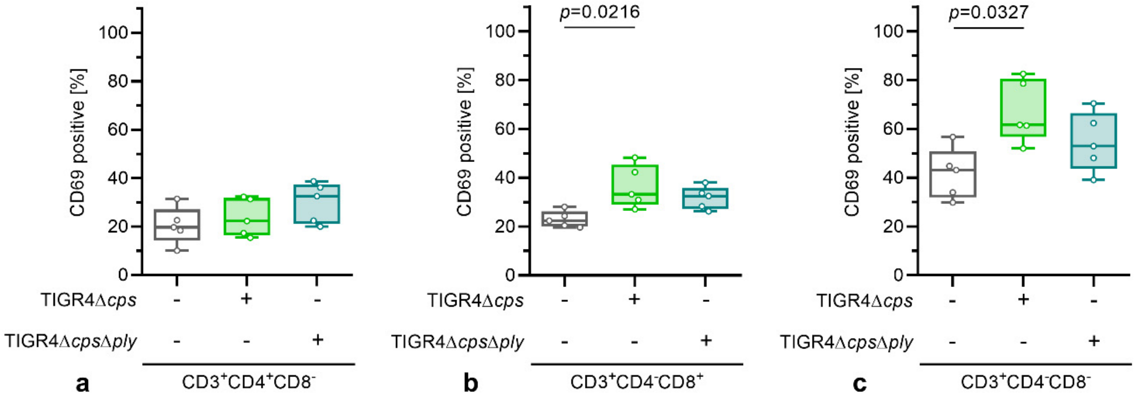

Figure S7 Activation of T cells in response to infected moDCs. Infected MoDCs were co-cultured with PBMCs for 3 days (n=5). T cell activation was evaluated assessing the frequencies of $CD69^+$ cells. The analysis was separated between $CD4^+$ (**a**), $CD8^+$ (**b**) and double negative (**c**) T cells. The data are displayed as box plots. The level of significance was determined using Kruskal-Wallis test with Dunn`s post-test.

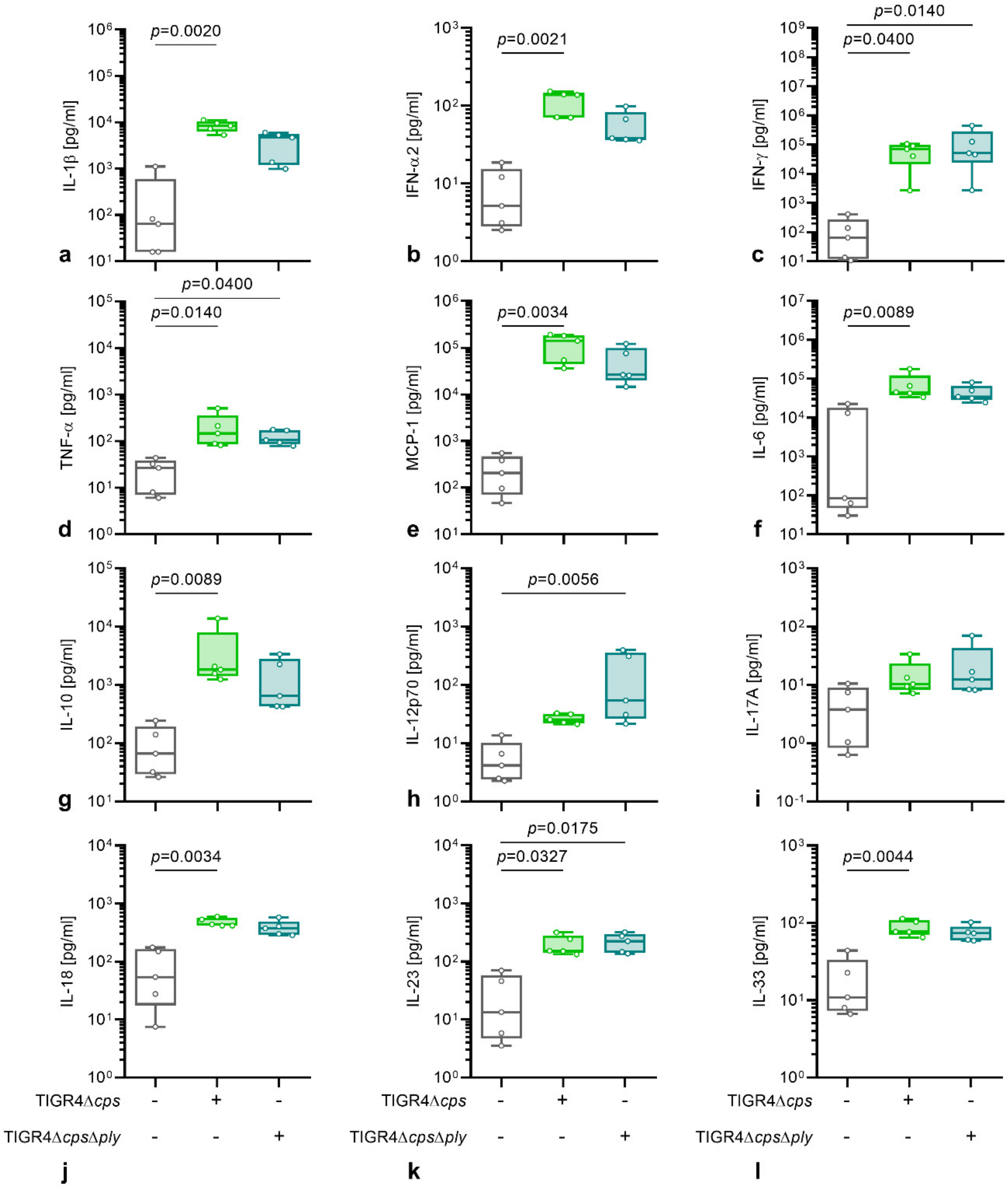

Figure S8 Cytokine secretion of PBMCs co-cultured with infected DCs was measured via a multiplex assay (n=5). The concentration of IL-1β (**a**), IFN-α2 (**b**), IFN-γ (**c**), TNF-α (**d**), MCP-1 (**e**), IL-6 (**f**), IL-10 (**g**), IL-12p70 (**h**), IL-17A (**i**), IL-18 (**j**), IL-23 (**k**) and IL-33 (**l**) was determined using flow cytometry. The data are displayed as box plots. The level of significance was determined using Kruskal-Wallis test with Dunn`s post-test.
